# The San Francisco Declaration on Research Assessment

**DOI:** 10.1242/dmm.012955

**Published:** 2013-05-20

**Authors:** Ross Cagan

**Affiliations:** Ross Cagan is at the Department of Developmental and Regenerative Biology and Graduate School of Biomedical Sciences, Icahn School of Medicine at Mount Sinai, New York, USA, e-mail: dmmeditor@biologists.com

On December 16, 2012, a group of editors and publishers of scholarly journals, including representatives from The Company of Biologists (COB), publisher of *Disease Models & Mechanisms*, gathered at the Annual Meeting of The American Society for Cell Biology in San Francisco, CA, USA to discuss current issues related to how the quality of research output is evaluated, and how the primary scientific literature is cited.

The impetus for the meeting was the consensus that impact factors for many cell biology journals do not accurately reflect the value to the cell biology community of the work published in these journals; this also extends to other fields in the biological sciences. The group therefore wanted to discuss how to better align measures of journal and article impact with journal quality.

There is also an alarming trend for the citation of reviews over primary literature, driven in part by space limitations that are imposed by some journals. Because this citation bias contributes to lower citation indices for journals that focus mainly on primary literature, the group discussed ways to combat this trend as well.

“...impact factors for many cell biology journals do not accurately reflect the value to the cell biology community of the work published in these journals”

The outcome of this meeting and further discussions is a set of recommendations that is referred to as the San Francisco Declaration on Research Assessment (DORA), published in May 2013. The recommendations are listed below, or you can read the entire Declaration at http://www.ascb.org/SFdeclaration.html.

The COB and its journals, *Disease Models & Mechanisms*, *Journal of Cell Science*, *Development*, *The Journal of Experimental Biology* and *Biology Open*, fully support this initiative. In concordance with the recommendations, all COB journals will provide their impact factors alongside a variety of other journal-based metrics; request an author contribution statement for all research articles; place no restrictions on the reuse of reference lists; and have no limitations on the number of references. The COB is also working with its online hosts, HighWire, to provide a range of article-level metrics.

It is The COB’s hope that this initiative will help to ensure that research assessment remains informed and fair.

## San Francisco DORA recommendations

### General recommendation

(1) Do not use journal-based metrics, such as journal impact factors, as a surrogate measure of the quality of individual research articles, to assess an individual scientist’s contributions, or in hiring, promotion or funding decisions.

### For funding agencies

(2) Be explicit about the criteria used in evaluating the scientific productivity of grant applicants and clearly highlight, especially for early-stage investigators, that the scientific content of a paper is much more important than publication metrics or the identity of the journal in which it was published.

(3) For the purposes of research assessment, consider the value and impact of all research outputs (including datasets and software) in addition to research publications, and consider a broad range of impact measures, including qualitative indicators of research impact such as influence on policy and practice.

### For institutions

(4) Be explicit about the criteria used to reach hiring, tenure and promotion decisions, clearly highlighting, especially for early-stage investigators, that the scientific content of a paper is much more important than publication metrics or the identity of the journal in which it was published.

(5) For the purposes of research assessment, consider the value and impact of all research outputs (including datasets and software) in addition to research publications, and consider a broad range of impact measures, including qualitative indicators of research impact such as influence on policy and practice.

### For publishers

(6) Greatly reduce the emphasis on the journal impact factor as a promotional tool, ideally by ceasing to promote the impact factor or by presenting it in the context of a variety of journal-based metrics (e.g. 5-year impact factor, EigenFactor, SCImago, *h*-index, editorial and publication times, etc.) that provide a richer view of journal performance.

(7) Make available a range of article-level metrics to encourage a shift towards assessment that is based on the scientific content of an article rather than on the publication metrics of the journal in which it was published.

(8) Encourage responsible authorship practices and the provision of information about the specific contributions of each author.

(9) Whether a journal is open-access or subscription-based, remove all reuse limitations on reference lists in research articles and make them available under the Creative Commons Public Domain Dedication licence.

(10) Remove or reduce the constraints on the number of references in research articles and, where appropriate, mandate the citation of primary literature in favour of reviews in order to give credit to the group(s) who first reported a finding.

### For organisations that supply metrics

(11) Be open and transparent by providing data and methods used to calculate all metrics.

(12) Provide the data under a licence that allows unrestricted reuse, and provide computational access to data, where possible.

(13) Be clear that inappropriate manipulation of metrics will not be tolerated; be explicit about what constitutes inappropriate manipulation and what measures will be taken to combat this.

(14) Account for the variation in article types (e.g. reviews versus research articles), and in different subject areas when metrics are used, aggregated or compared.

### For researchers

(15) When involved in committees making decisions about funding, hiring, tenure or promotion, make assessments based on scientific content rather than on publication metrics.

(16) Wherever appropriate, cite primary literature in which observations are first reported rather than reviews in order to give credit where credit is due.

(17) Use a range of article metrics and indicators in personal/supporting statements as evidence of the impact of individual published articles and other research outputs.

(18) Challenge research assessment practices that rely inappropriately on journal impact factors, and promote and teach best practice that focuses on the value and influence of specific research outputs.

